# Absence of antibodies against KIR4.1 in multiple sclerosis: A three-technique approach and systematic review

**DOI:** 10.1371/journal.pone.0175538

**Published:** 2017-04-17

**Authors:** Miquel Navas-Madroñal, Ana Valero-Mut, María José Martínez-Zapata, Manuel Javier Simón-Talero, Sebastián Figueroa, Nuria Vidal-Fernández, Mariana López-Góngora, Antonio Escartín, Luis Querol

**Affiliations:** 1 Multiple Sclerosis Unit, Department of Neurology, Hospital de la Santa Creu i Sant Pau, Universitat Autònoma de Barcelona, Barcelona, Spain; 2 Iberoamerican Cochrane Centre, Biomedical Research Institute Sant Pau (IIB Sant Pau), CIBER Epidemiologia y Salud pública (CIBERESP), Barcelona, Spain; Medizinische Universitat Innsbruck, AUSTRIA

## Abstract

**Introduction:**

Antibodies targeting the inward-rectifying potassium channel KIR4.1 have been associated with multiple sclerosis (MS) but studies using diverse techniques have failed to replicate this association. The detection of these antibodies is challenging; KIR4.1 glycosylation patterns and the use of diverse technical approaches may account for the disparity of results. We aimed to replicate the association using three different approaches to overcome the technical limitations of a single technique. We also performed a systematic review to examine the association of anti-KIR4.1 antibodies with MS.

**Methods:**

Serum samples from patients with MS (n = 108) and controls (n = 77) were tested for the presence of anti-KIR4.1 antibodies using three methods: 1) by ELISA with the low-glycosylated fraction of recombinant KIR4.1 purified from transfected HEK293 cells according to original protocols; 2) by immunocytochemistry using KIR4.1-transfected HEK293 cells; and 3) by immunocytochemistry using the KIR4.1.-transfected MO3.13 oligodendrocyte cell line. We developed a systematic review and meta-analysis of the association of anti-KIR4.1 antibodies with MS according to the Preferred Reporting Items for Systematic Reviews and Meta-Analyses (PRISMA) guidelines.

**Results:**

We did not detect anti-KIR4.1 antibodies in the MS patients or in controls using ELISA. Neither did we detect any significant reactivity against the antigen on the cell surface using the KIR4.1-transfected HEK293 cells or the KIR4.1-transfected MO3.13 cells. We included 13 prospective controlled studies in the systematic review. Only three studies showed a positive association between anti-KIR4.1 and MS. Clinical and statistical heterogeneity between studies precluded meta-analysis of their results.

**Conclusion:**

We found no association between anti-KIR4.1 antibody positivity and MS. Although this lack of replication may be due to technical limitations, evidence from our study and others is mounting against the role of KIR4.1 as a relevant MS autoantigen.

## Introduction

Multiple sclerosis (MS) is an autoimmune and neurodegenerative disease of the central nervous system that arises in genetically susceptible individuals exposed to environmental risk factors[1]. Clinical, genetic and experimental data in MS suggest a primary autoimmune response against oligodendrocytes is followed by a secondary neurodegenerative process that leads to disability[2]. Although MS is generally considered a T-cell mediated disease, B-cells populate the demyelinating lesions and the cerebrospinal fluid (CSF) and form lymphoid follicles in the brain parenchyma and the meninges[3]. Importantly, the presence of oligoclonal bands in the CSF, the most frequently observed lab abnormality, is useful for diagnosis and MS conversion risk stratification[4,5]. Moreover, B-cell depletion has proven an effective treatment for MS, suggesting B-cells play a central role in the pathogenesis of MS [6]. The search for the target antigen(s) of the immune response is an active field of MS research[7]. The discovery of antibodies targeting aquaporin-4 provided a biological marker to classify these patients as a specific disease subset (neuromyelitis optica) and the proof of principle that humoral factors could also mediate MS pathogenesis[8]. Despite exhaustive research using diverse approaches, the target antigen (or antigens) of the MS immune response remains largely unknown. Thus, MS lacks a disease-specific diagnostic biomarker and diagnosis relies on clinical and brain imaging criteria[9]. In 2012, Srivastava et al published a landmark article describing the presence of antibodies against the inward-rectifying potassium channel 4.1 (KIR4.1) in approximately half of the MS patients they studied [10]. A similar study in children with MS by the same group yielded similar results[11]. Antibodies against KIR4.1 were originally described using ELISA assays in which either whole protein KIR4.1 or KIR4.1 extracellular loop peptides served as substrate antigens, but subsequent studies failed to replicate the peptide ELISA results[12–15]. Lennon et al failed to replicate the original report using a cell-based assay with KIR4.1-transfected human embryonic kidney (HEK) cells[16]. KIR4.1 is present in diverse tissues and cell-types, including oligodendrocytes, where it is expressed as KIR4.1 homotetramers, and astrocytes, where it forms heterotetramers with KIR5.1[17]. Furthermore, KIR4.1 glycosylation varies depending on the cell type and the whole protein ELISA results are heavily influenced by the glycosylation status of the protein[18]. These KIR4.1 features could account for the conflicting results, prompting replication studies using the original ELISA system and addressing the influence of glycosylation patterns. Using the original ELISA system, two independent groups recently published reports that fail to replicate KIR4.1 as a frequent autoantigen in MS[19,20].

Our study aims to solve the controversy using a four-fold approach. Experimentally, we used a whole-KIR4.1 ELISA to try to replicate the results reported in a former study and two cell-based assays. One assay used KIR4.1-transfected HEK cells to display KIR4.1 in non-denaturating conditions on the surface of cells used to express and purify the protein for the ELISA test. The other used the KIR4.1-transfected MO3.13 cell line to display the KIR4.1 on a cell type that would replicate the natural glycosylation patterns of KIR4.1 better than HEK cells. Finally, we systematically reviewed the evidence on the association of MS with anti-KIR4.1 antibodies and attempted a meta-analysis to help solve this controversy.

## Methods

### Subjects, informed consent and protocol approvals

Consecutive patients fulfilling the McDonald diagnostic criteria for MS[9] and visited in our Unit from June 2014 to June 2016 were included in the study. Controls (n = 77) consisted of sera from healthy subjects (NC; n = 13) and from subjects with other neuroinflammatory and degenerative disorders: multifocal motor neuropathy (NMM; n = 16); chronic inflammatory demyelinating polyneuropathy (CIDP; n = 24); myasthenia gravis (MG; n = 16); amyotrophic lateral sclerosis (ALS; n = 8). Serum samples were aliquoted and stored at -80°C until needed. All patients gave written informed consent to participate in the study under a protocol approved by the Ethics Committee at Hospital de la Santa Creu i Sant Pau (protocol IIBSP-KIR-2014-12). Sera from patients and controls were collected in two different sample collections following Instituto de Salud Carlos III protocols for sample collection (C.0003085, for patients; C.0002365, courtesy of Professor Isabel Illa, for controls)

### Enzyme-linked immunosorbent assay (ELISA)

HEK293 cells were cultured in 100 mm culture dishes in 5% fetal bovine serum-supplemented DMEM medium plus penicillin-streptavidin, sodium-pyruvate and L-glutamine. At 70% cell confluence, the mammalian expression vector pcDNA3.1 encoding full-length human KIR4.1 with a His-tag in the C-terminus)[20] was transfected with Lipofectamine 2000 (Thermo-Fisher, Spain) and incubated at 37°C. After 24 hours, cells were washed with PBS, centrifuged and KIR4.1 purified following the original Srivastava et al[10] protocol. Briefly, cells were lysed, centrifuged and KIR4.1 protein purified using the HisPur Cobalt resin (Thermo Fisher, Spain). ELISA was performed as described by Srivastava et al. using a final concentration of 7ug per milliliter of elution fraction 3 (EF3), in which low-glycosylated KIR4.1 oligomers are found. Elution fraction 1 reactivity was used as the background control for each sample. Results were read using a Beckman AD340 plate reader (Beckman-Coulter, Indianapolis, IN). Results were represented as the difference of the elution fraction 3 minus elution fraction 1 (EF1) optical density (OD). Samples were considered positive by ELISA when they had a ΔOD higher than mean healthy control ΔOD plus five standard deviations. All samples were tested at the same time and only analyzed if the ELISA fulfilled the quality criteria proposed by Srivastava[18] and Marnetto et al[21]: ELISA signal of the rat monoclonal anti-KIR4.1 antibody 20F9 (courtesy of Srivastava and Hemmer) with an OD lower than 0,36, EF3 OD higher than 1,361 and assay diluent OD lower than 0,1 were the quality criteria used to proceed with ELISA analysis. Positive control samples were used to help set-up the assay (kindly provided by Rajneesh Srivastava)

Sensitivity and specificity of the KIR4.1 ELISA and their respective confidence intervals were calculated. The non-parametric Kruskal Wallis test with post-hoc multiple comparisons test was used to compare average OD between groups. All statistical analyses were performed with GraphPad Prism v5.0.

### Immunocytochemistry (ICC)

KIR4.1-transfected MO3.13 oligodendrocyte cell line (Cedarlane Labs, Canada) and HEK293 cells were used for ICC experiments. Briefly, HEK293 cells or the MO3.13 oligodendrocyte cell line were cultured in 60mm culture plates with poly-D-Lysine-coated coverslips and transfected as previously described. After 24 hours, cells were washed with PBS, fixed with 4% paraformaldehyde and blocked with 5% goat serum in PBS for 1 hour. Coverslips were then incubated with patients’ sera at 1:100 in blocking buffer and with a 1:500 commercial anti-KIR4.1 antibody (Millipore AB8518) at room temperature. After 1 hour, cells were washed with PBS and incubated with Alexa-Fluor goat-anti-human 594nm and goat anti-rabbit 488nm (Thermo-Scientific, Spain) secondary antibodies diluted 1:500. Finally, coverslips were washed and mounted using Vectashield with DAPI (Vector Labs, UK). Evaluators (LQ, MN) were blinded for the diagnosis. Demonstration of KIR4.1 expression on MO3.13 cells was performed in an independent experiment described in Supplementary Methods.

### Systematic review

**Fig 1:**
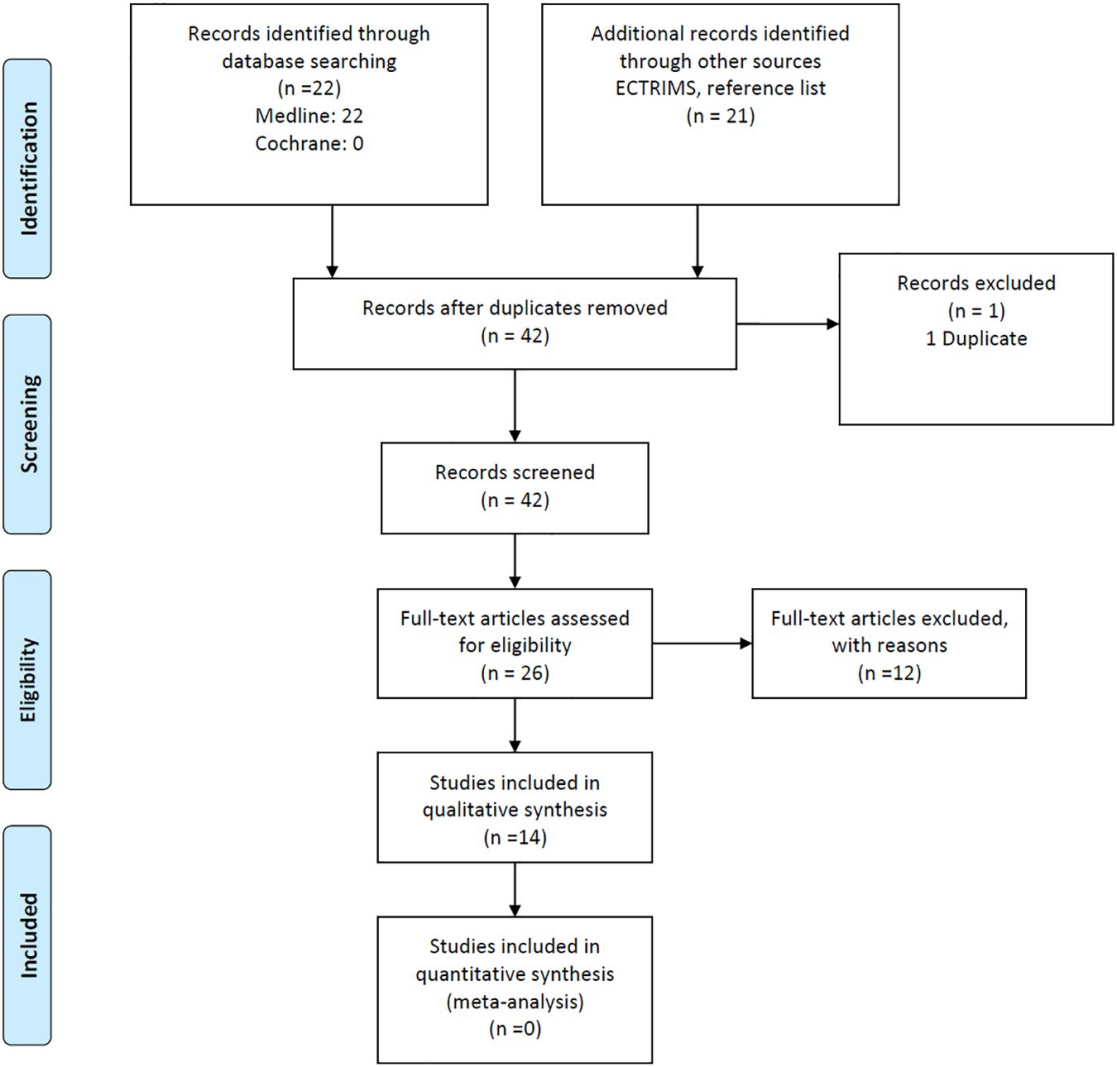
Systematic review flowchart. Thirteen studies addressing the prevalence of anti-KIR4.1 antibodies in MS and controls were identified. These 13 studies plus our own results were used for the systematic review.

We aimed to assess the association of anti-KIR4.1 antibodies with MS. We proceeded according to the Preferred Reporting Items for Systematic Reviews and Meta-Analyses (PRISMA) guidelines [22]. We included studies published in English or Spanish, addressing anti-Kir4.1 antibody positivity in patients with MS diagnosed according to the 2010 McDonald criteria compared to healthy controls. Exclusion criteria included lack of outcomes of interest in the report (Fig 1). The search strategy included the Cochrane Central Register of Controlled Trials, Ovid Medline (1946 to August 2016) and the European Committee for Treatment and Research in MS (ECTRIMS) abstract databases. The medical subject heading (MeSH) terms and key words used in the search included the terms: “multiple sclerosis AND Kir4.1” and ("multiple sclerosis"[MeSH Terms] OR ("multiple"[All Fields] AND "sclerosis"[All Fields]) OR "multiple sclerosis"[All Fields]) AND kir4.1[All Fields]. For data extraction, one author (AV) selected the studies, and two authors (LQ, AV) independently assessed each study to check its eligibility. References meeting the inclusion criteria were retrieved in full and further assessed independently by the same two review authors. Data in each study were extracted by two independent reviewers (LQ and AV) using the same standardized method. We contacted study authors by e-mail to obtain additional information when data were missing or unclear. The methodological quality was assessed by the Effective Public Health Practice Project’s Quality Assessment Tool (http://www.ephpp.ca/tools.html), which has six components: selection bias, study design, confounders, blinding, data collection methods, and withdrawals/drop-outs. Each component was classified as strong, moderate or weak. A global methodological quality assessment was obtained according to the number of components rated as weak (0, 1, or >1) as strong, moderate or weak[23]. The information extracted was: publication data, study design, patients’ characteristics, and patients with positivity against anti-KIR4.1 antibodies. When two or more different assays yielded different frequencies of positive anti-KIR4.1 antibody results in the same report, the standard test (ELISA) was chosen for analysis. The primary outcome was percentage of study participants with positive anti-KIR4.1 antibodies. The comparison was MS patients versus controls. The odds ratios (OR) and 95% confidence interval (CI) were calculated to compare groups. We used a random effect model[24] for all analyses, as we expected variation in effects due to differences in study populations and methods to measure anti-KIR4.1 antibodies. Heterogeneity between studies was evaluated using the I^2^ statistic, categorized as follows: <30% not important; 30%-50% moderate; 50%-75% substantial; and 75%-100% considerable[25]. The data were not pooled if I^2^ was over 75%. Statistical software used was Review Manager 5.3[26].

## Results

One hundred and eight MS patients were included in the study and tested by ELISA and ICC with both KIR4.1-transfected HEK293 cells and the KIR4.1-transfected MO3.13 cell line. S1 Table shows basic demographic features of patients and controls.

### ELISA

**Fig 2:**
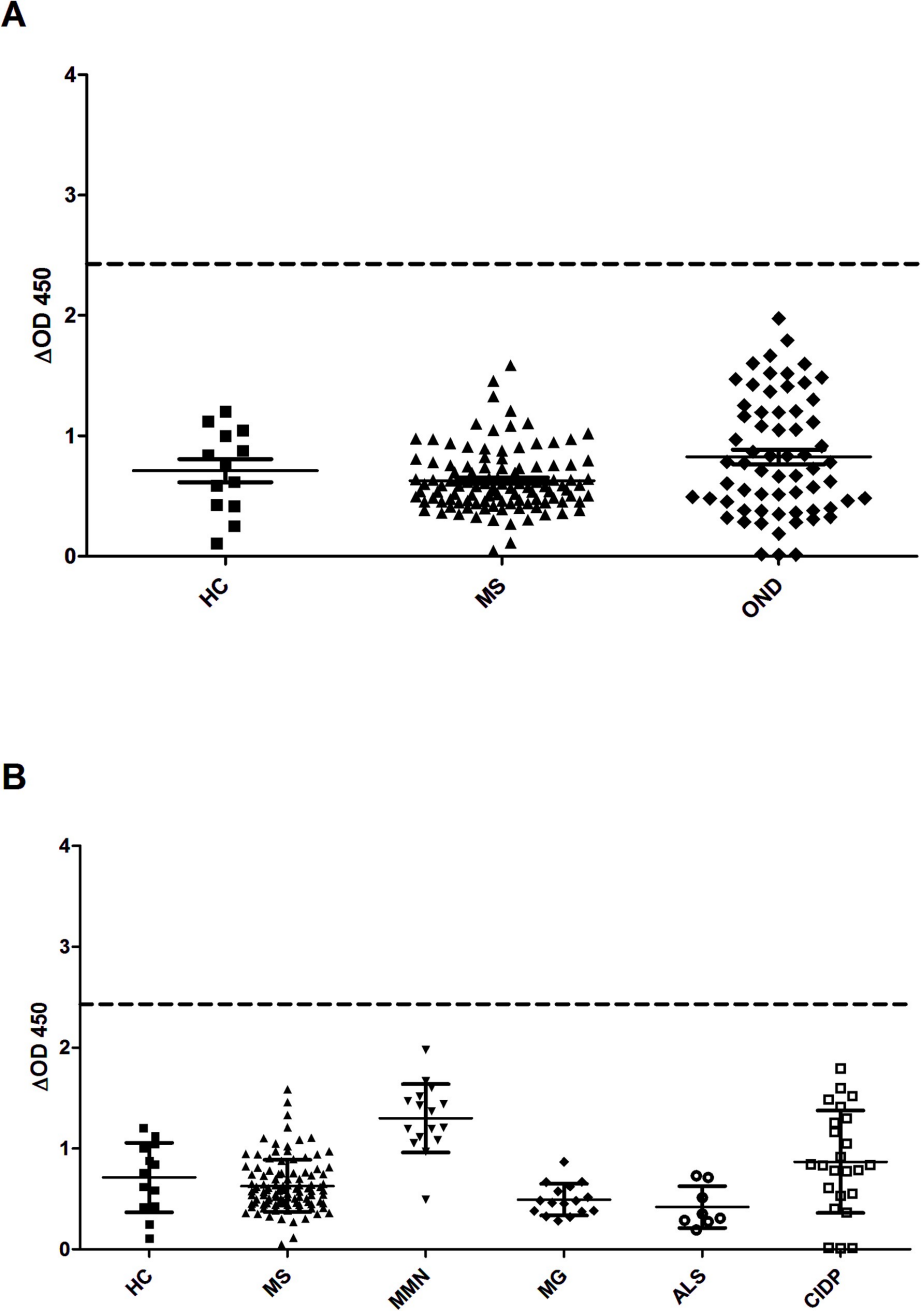
IgG reactivity against KIR4.1 does not differ in MS and controls by ELISA. Low-glycosylated fraction of the KIR4.1 whole protein complex was purified and used to detect anti-KIR4.1 antibodies by ELISA. None of the 108 MS patients or disease controls reached a ΔOD signal above healthy control average ΔOD plus 5 SD. The average ΔOD signal did not show significant differences between groups (A). The average ΔOD signal from patients with MMN was significantly higher (p<0.0001, Kruskal-Wallis) than that from any other group except CIDP. The CIDP average ΔOD was higher than the average in the ALS and MG groups (p<0.0001, Kruskal-Wallis; Fig 2B).

None of the 108 MS patients or the healthy and disease controls reached a ΔOD higher than average healthy control ΔOD plus 5 standard deviations and, thus, failed to be considered positive by ELISA according to the pre-established criteria (Fig 2A). The sensitivity of the assay to detect MS patients in comparison with any type of control was 0.000 (0.000 to 3.535; 95% confidence interval) and specificity was 1 (0.952 to 1, 95% confidence interval). Comparison of the average ΔOD signal between groups did not differ between MS patients and healthy participants or disease controls (p = 0.08, Kruskal-Wallis). The average ΔOD signal from patients with MMN was significantly higher than in any other group except CIDP (p<0.0001, Kruskal-Wallis). The CIDP average ΔOD was higher than that in the ALS and MG groups (Fig 2B; p<0.0001, Kruskal-Wallis).

### Immunocytochemistry

**Fig 3:**
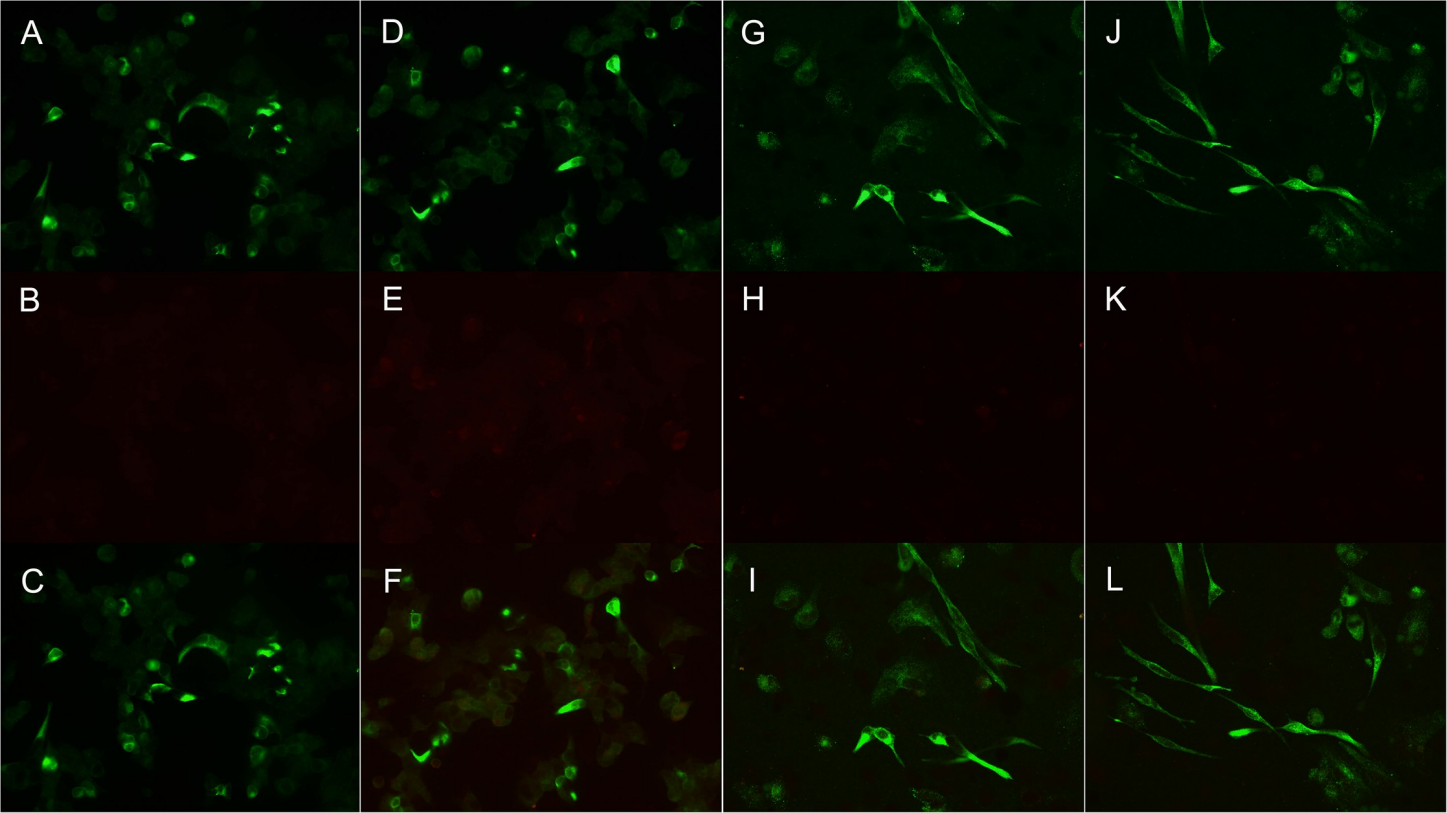
Lack of identification of KIR4.1 antibodies in serum samples using ICC. MS patients or disease controls did not show antibodies against KIR4.1 tested by ICC in KIR4.1 transfected HEK293 (A-F) or MO3.13 (G-L). Anti-KIR4.1 commercial antibody reactivity is shown in the upper row (A, D, G, J), serum IgG reactivity from an MS patient (B and H) and from a disease control (E and K) in the middle row, and merge images (C, F, I, L) in the lower row. The MS patient and disease control showing highest ΔOD were chosen to assemble this Figure.

We performed ICC experiments to further explore the presence of anti-KIR4.1 antibodies in MS patients. All patients and disease controls tested negative by immunocytochemistry over KIR4.1-transfected HEK293 cells despite abundant surface expression of KIR4.1 (Fig 3A–3F). Considering the possibility that glycosylation patterns could differ between HEK293 and oligodendrocytes, we repeated the ICC experiments using KIR4.1-transfected MO3.13 cells[18]. No MS patient or disease control testing was positive for surface staining on MO3.13 cells, despite efficient KIR4.1 transfection and cell surface expression (Fig 3G–3L). Fig 3 shows patients and disease controls with the highest ΔOD signals by ELISA.

### Systematic review

**Fig 4:**
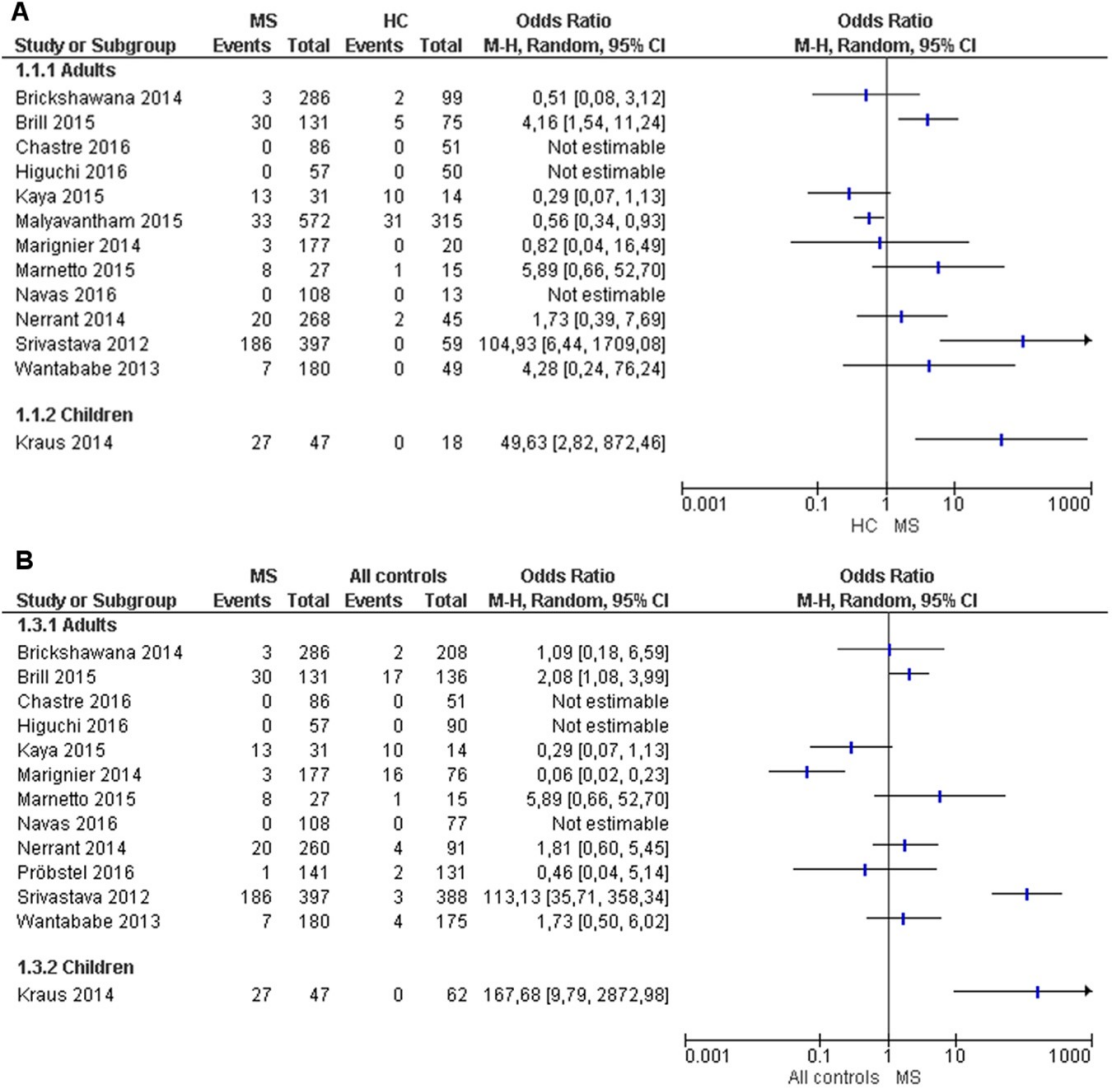
Forest plot. Thirteen studies were included in the systematic review. Three of them showed evidence of an association of KIR4.1 with MS patients compared to healthy controls (A) or all controls (B). One showed a weak association of KIR4.1 antibodies with healthy controls. None of the other reports found any association of KIR4.1 antibodies with MS, healthy or disease controls. The study by Probstel[19] was excluded from Fig 4A forest plot because it lacked comparison with healthy controls, as specified in the inclusion criteria (A). The study by Malyavantham[15] was excluded from Fig 4B forest plot because it lacked comparison with disease controls (B).

Thirteen studies were included, 12 in adult subjects and one in children (Fig 1 and S1 Fig). The studies were developed in the USA (3)[15,16,20], Germany (2)[10,11], France (2)[12,27], Japan (2)[28,29], Israel (1)[14], Italy (1)[21], Spain (1), Switzerland (1)[19], and Turkey (1)[13]. They showed substantial variability in the technical determination of anti-KIR4.1 and control group populations. The methodological quality was qualified as week in five studies[13,21,27–29], moderate in four studies[11,16,20,30] plus our own, and strong in three studies[10,14,15]. One study[19] was not included due to lack of normal controls (S1 Fig). Compared with studies in healthy controls, three studies in MS patients showed an association of positivity of anti-KIR4.1[10,11,14] and seven studies, including our own, showed no significant differences[12,13,16,20,21,27–29] (Fig 4). The statistical heterogeneity between studies was over 75% and did not allow data pooling to perform meta-analysis.

## Discussion

We found no differences in the proportion of MS patients harboring autoantibodies against KIR4.1 compared to disease and healthy controls using the same ELISA-based detection assay described in the original report and using two different cell-based assays[10,19,20].

The description for clinically useful biomarkers is of special relevance in MS[31].MS is a chronic, heterogeneous and disabling disease that mainly affects young adults. It is diagnosed based on clinical and radiological criteria but diagnostic uncertainty often persists in the initial phases. Moreover, there is clinical, pathological, experimental and genetic evidence that B-cells and autoantibodies play a role in MS pathogenesis[32]. For these reasons, the quest for autoantibodies is a topic of great relevance in MS research. The description of anti-KIR4.1 antibodies was a potential landmark discovery[33], first, because KIR4.1 was observed to co-localize with aquaporin-4 in the brain and represented a plausible antigen[34] and, second, because it provided the first solid evidence that disease-specific autoantibodies could be found in a substantial proportion of adult MS patients[10]. This first report was followed by a second study by the same group describing a similar proportion of positive patients among children with MS[11]. Importantly, these studies did not identify clinical, radiological or therapeutic features that were specifically associated with the presence of anti-KIR4.1 antibodies in MS patients. The results from early replication studies using the KIR4.1 extracellular loop (residues 83–120) peptide to test anti-KIR4.1 antibodies by ELISA were insufficiently consistent to demonstrate a clear association of anti-KIR4.1 and MS even though the same detection assay was used[12,27]. A thorough replication study including a multimodal approach also failed to confirm the association of anti-KIR4.1 antibodies with MS[16]. These reports did not use the full-length KIR4.1 protein ELISA for anti-KIR4.1 antibody detection, and they did not take into consideration the existence of KIR4.1 oligomers with different degrees of glycosylation that were reported to influence the ELISA results[18]. At the 2015 ECTRIMS Congress, Marnetto et al presented a small study in which they addressed the technical difficulties associated with anti-KIR4.1 testing and partially replicated the results of the original report[21]. However, this presentation highlighted “session-specific” success in detecting the antibodies, further highlighting the inconsistent results. Recently, two replication studies by Chastre et al and Probstel et al, using the full KIR4.1 complex and addressing the technical caveats of the assay, did not show differences in anti-KIR4.1 antibodies between patients and healthy or disease controls[19,20]. Other authors, using unbiased approaches to detect autoantibodies with protein arrays, and not specifically aimed to detect anti-KIR4.1 antibodies, also failed to identify KIR4.1 as a candidate antigen, but the expression systems, experimental designs and antigen sources on the arrays for these studies are significantly different to the original description[35,36].

In this context, we developed the present study to specifically address the technical differences that could lead to inconsistent results in the association of anti-KIR4.1 antibodies with MS. First, we used the full-length KIR4.1 protein in an ELISA using methods as described in the original report and in two recent studies[19,20], and we found no differences in the proportion of patients or controls testing positive for these antibodies. On the contrary, the average ELISA signal tended to be higher in specific types of neuroimmune controls, such as MMN and CIDP. This observation is in agreement with findings in a recent study[19] in which anti-KIR4.1 reactivity correlated significantly with reactivity against sham antigens, suggesting that higher nonspecific antibody promiscuity, independently of the disorder, could result in higher anti-KIR4.1 reactivity[19]. Second, we transfected KIR4.1 in HEK293 cells using the same mammalian expression vector (pDNA3.1+) that Srivastava et al, Chastre et al and ourselves had used for protein purification. ELISA allowed us to detect the low-glycosylated isoforms using the Srivastava and Marnetto criteria with the rat 20F9 antibody but we were unable to identify surface staining in KIR4.1-transfected HEK293 cells even in patients and controls showing the highest ELISA signal. If low-glycosylated fractions of KIR4.1 are expressed in HEK293 cells and are detectable by ELISA, sera from MS patients should react against the surface of these cells, but we did not find any hint of reactivity. Brickshawana et al attempted to demonstrate surface staining of sera from MS patients against KIR4.1-transfected HEK293 cells [16]. However, although they demonstrated functional activity of the potassium channel and correct expression at the surface, KIR4.1 was encoded in the pEGFP-C2 expression vector. Expression of the fusion protein (KIR4.1-GFP) could, in theory, lead to epitope modifications and ultimately be the source of inconsistency with the original report. Finally, we performed ICC using patient sera over the MO3.13 human cell line, transfected with KIR4.1, and here again we failed to detect any relevant anti-KIR4.1 reactivity. The MO3.13 cell line was originated from the fusion of a human rhabdomyosarcoma line and adult human oligodendrocytes obtained from a surgical specimen. MO3.13 cells show features of immature oligodendrocytes and, under certain conditions, they can differentiate and express myelin proteins[37]. In fact, they are used as an in vitro model for human oligodendrocytes[38] and have been used for autoantibody screening in MS patients [39]. MO3.13 cells presumably recapitulate mature oligodendrocyte glycosylation patterns better than HEK293 cells. Therefore, the KIR4.1 protein expressed on the MO3.13 surface should be a better antigen to detect relevant anti-KIR4.1 antibodies. Again, we did not find any surface staining in any patient or control using this system, ruling out a significant influence of the cell system in our KIR4.1-transfected HEK293 assay. There remains the possibility that the hybrid origin of the MO3.13 cells influences the glycosylation patterns of KIR4.1 and, thus the ability to detect anti-KIR4.1 antibodies in this system.

Finally, we developed a systematic review with the ultimate objective of performing a meta-analysis on the usefulness of anti-KIR4.1 antibodies. We included 13 studies with diverse methodological quality. The main methodological weakness of studies is that confounders were not considered (e.g. matching case and controls by age and sex) in the design or analysis. A publication bias was detected, with smaller studies showing no-association with anti-KIR4.1 and MS (S2 Fig). The meta-analysis was not performed, however, because the calculation of an I^2^ test over the 75% limit showed high heterogeneity among the studies included.

In summary, we failed to identify anti-KIR4.1 antibodies in patients with MS using the full-length, low-glycosylated KIR4.1 ELISA assay and HEK293 and oligodendrocyte cell-based assays. Although technical caveats are still a matter of concern to detect antibodies targeting this complicated antigen, using very diverse approaches, evidence is accumulating against the confirmation of KIR4.1 as a relevant MS autoantigen.

## Supporting information

S1 PRISMA Checklist PRISMA checklist disclosing information necessary for the standardization of the systematic review. (DOC)

S1 Fig This table summarizes all studies included in the systematic review and displays information about date and country of publication, n of patients and controls, technique used to detect anti-KIR4.1 antibodies, the study design and their methodological quality according to systematic review guidelines. MS: multiple sclerosis, OND: other neurological diseases, HC; healthy controls.
(PDF)

S2 Fig Funnel plot comparison: Multiple sclerosis vs healthy people, outcome: Positive anti-KIR4.1. The funnel plot detects a publication bias, with smaller studies showing no-association with anti-KIR4.1 and MS.
(PDF)

S1 Supplementary Methods Description of additional immunocytochemistry experiments to demonstrate surface expressionof recombinant Kir4.1 in transfected MO3.13 cells. (PDF)

S1 Table Basic demographic features of patients with MS and controls included in the study. Epidemiological data other than gender were unavailable for 15 disease controls and average age reflects that of the ones in which information was available. MS: multiple sclerosis; HC: Healthy controls; OND: Other neurological diseases; RR: relapsing-remitting; SP: secondary progressive; PP: primary progressive.
(DOCX)

## References

[1] Ransohoff RM, Hafler D a., Lucchinetti CF. Multiple sclerosis-a quiet revolution. Nat Rev Neurol. 2015;11: 134–42. 25686758 10.1038/nrneurol.2015.14 PMC4556342

[2] Nylander A, Hafler DA. Multiple sclerosis. J Clin Invest. 2012;122: 1180–8. doi: 10.1172/JCI58649 22466660 10.1172/JCI58649 PMC3314452

[3] Disanto G, Morahan JM, Barnett MH, Giovannoni G, Ramagopalan S V. The evidence for a role of B cells in multiple sclerosis. Neurology. 2012;78: 823–832. doi: 10.1212/WNL.0b013e318249f6f0 22411958 10.1212/WNL.0b013e318249f6f0 PMC3304944

[4] Villar LM, Sádaba MC, Roldán E, Masjuan J, González-Porqué P, Villarrubia N, et al Intrathecal synthesis of oligoclonal IgM against myelin lipids predicts an aggressive disease course in MS. J Clin Invest. 2005;115: 187–94. doi: 10.1172/JCI22833 15630459 10.1172/JCI22833 PMC539201

[5] Villar LM, Casanova B, Ouamara N, Comabella M, Jalili F, Leppert D, et al Immunoglobulin M oligoclonal bands: Biomarker of targetable inflammation in primary progressive multiple sclerosis. Ann Neurol. 2014;76: 231–40. doi: 10.1002/ana.24190 24909126 10.1002/ana.24190

[6] Hauser SL, Waubant E, Arnold DL, Vollmer T, Antel J, Fox RJ, et al B-cell depletion with rituximab in relapsing-remitting multiple sclerosis. N Engl J Med. 2008;358: 676–88. doi: 10.1056/NEJMoa0706383 18272891 10.1056/NEJMoa0706383

[7] Fraussen J, Claes N, de Bock L, Somers V. Targets of the humoral autoimmune response in multiple sclerosis. Autoimmun Rev. Elsevier B.V.; 2014;13: 1126–37. doi: 10.1016/j.autrev.2014.07.002 25108168 10.1016/j.autrev.2014.07.002

[8] Lennon PVA, Wingerchuk DM, Kryzer TJ, Pittock SJ, Lucchinetti CF, Fujihara K, et al A serum autoantibody marker of neuromyelitis optica: Distinction from multiple sclerosis. Lancet. 2004;364: 2106–2112. doi: 10.1016/S0140-6736(04)17551-X 15589308 10.1016/S0140-6736(04)17551-X

[9] Polman CH, Reingold SC, Banwell B, Clanet M, Cohen J a, Filippi M, et al Diagnostic criteria for multiple sclerosis: 2010 revisions to the McDonald criteria. Ann Neurol. 2011;69: 292–302. doi: 10.1002/ana.22366 21387374 10.1002/ana.22366 PMC3084507

[10] Srivastava R, Aslam M, Kalluri SR, Schirmer L, Buck D, Tackenberg B, et al Potassium channel KIR4.1 as an immune target in multiple sclerosis. N Engl J Med. 2012;367: 115–23. doi: 10.1056/NEJMoa1110740 22784115 10.1056/NEJMoa1110740 PMC5131800

[11] Kraus V, Srivastava R, Kalluri SR, Seidel U, Schuelke M, Schimmel M, et al Potassium channel KIR4.1-specific antibodies in children with acquired demyelinating CNS disease. Neurology. 2014;82: 470–3. doi: 10.1212/WNL.0000000000000097 24415573 10.1212/WNL.0000000000000097

[12] Nerrant E, Salsac C, Charif M, Ayrignac X, Carra-Dalliere C, Castelnovo G, et al Lack of confirmation of anti-inward rectifying potassium channel 4.1 antibodies as reliable markers of multiple sclerosis. Mult Scler. 2014;20: 1699–703. doi: 10.1177/1352458514531086 24756568 10.1177/1352458514531086

[13] Kaya D, İdiman E, Karabay N, Altun Z, Mehdiyev Z.. Existence and significance of antibody against the inward rectifying potassium channel KIR4.1 in patients with multiple sclerosis in western part of Turkey. In: ECTRIMS Online Library 2015 p. 115171.

[14] Brill L, Goldberg L, Karni A, Petrou P, Abramsky O, Ovadia H, et al Increased anti-KIR4.1 antibodies in multiple sclerosis: could it be a marker of disease relapse? Mult Scler. 2015;21: 572–9. doi: 10.1177/1352458514551779 25392324 10.1177/1352458514551779

[15] Malyavantham K, Weinstock-Guttman B, Suresh L, Zivadinov R, Shanahan T, Badgett D, et al Humoral Responses to Diverse Autoimmune Disease-Associated Antigens in Multiple Sclerosis. PLoS One. 2015;10: e0129503 doi: 10.1371/journal.pone.0129503 26065913 10.1371/journal.pone.0129503 PMC4466031

[16] Brickshawana A, Hinson SR, Romero MF, Lucchinetti CF, Guo Y, Buttmann M, et al Investigation of the KIR4.1 potassium channel as a putative antigen in patients with multiple sclerosis: a comparative study. Lancet Neurol. Elsevier Ltd; 2014;13: 795–806. doi: 10.1016/S1474-4422(14)70141-3 25008548 10.1016/S1474-4422(14)70141-3 PMC4144430

[17] Schirmer L, Srivastava R, Kalluri SR, Böttinger S, Herwerth M, Carassiti D, et al Differential loss of KIR4.1 immunoreactivity in multiple sclerosis lesions. Ann Neurol. 2014; 1–55. 10.1002/ana.24168 24777949

[18] Srivastava R. Differential glycosylation of KIR4.1 in glia cells affects binding of autoantibodies in multiple sclerosis. In: ECTRIMS Online Library 2014 p. 64326.

[19] Pröbstel A-K, Kuhle J, Lecourt A-C, Vock I, Sanderson NSR, Kappos L, et al Multiple Sclerosis and Antibodies against KIR4.1. N Engl J Med. 2016;374: 1496–8. doi: 10.1056/NEJMc1507131 27074084 10.1056/NEJMc1507131

[20] Chastre A, Hafler DA, O’Connor KC. Evaluation of KIR4.1 as an Immune Target in Multiple Sclerosis. N Engl J Med. 2016;374: 1495–1496. doi: 10.1056/NEJMc1513302 27074083 10.1056/NEJMc1513302 PMC4918464

[21] Marnetto F, Valentino P, Caldano M, Bertolotto A. Anti-KIR4.1 antibodies as a biomarker in multiple sclerosis: problems and preliminary data. In: ECTRIMS Online Library 2015 p. 116353.

[22] Moher D, Liberati A, Tetzlaff J, Altman DG, PRISMA Group. Preferred reporting items for systematic reviews and meta-analyses: the PRISMA statement. PLoS Med. 2009;6: e1000097 doi: 10.1371/journal.pmed.1000097 19621072 10.1371/journal.pmed.1000097 PMC2707599

[23] Thomas BH, Ciliska D, Dobbins M, Micucci S. A process for systematically reviewing the literature: providing the research evidence for public health nursing interventions. Worldviews evidence-based Nurs. 2004;1: 176–84. 10.1111/j.1524-475X.2004.04006.x 17163895

[24] DerSimonian R, Laird N. Meta-analysis in clinical trials. Control Clin Trials. 1986;7: 177–88. Available: http://www.ncbi.nlm.nih.gov/pubmed/3802833 3802833 10.1016/0197-2456(86)90046-2

[25] Deeks JJ, Higgins JPT, Altman DG. on behalf of the Cochrane Statistical Methods Group (editors). Chapter 9: Analysing data and undertaking meta-analyses. Cochrane Handb Syst Rev Interv Version. 2009;5.

[26] Collaboration NCCTC, others. Review Manager (RevMan)[Computer program] Version 53. Copenhagen Nord Cochrane Centre, Cochrane Collab. 2014;

[27] Marignier R, Ruiz A, Benetollo C, Cavagna S, Vukusic S, Giraudon P. Potassium channel KIR4.1: a novel target for Neuromyelitis Optica antibodies? In: ECTRIMS Online Library 2014 p. 64827.

[28] Higuchi O, Nakane S, Sakai W, Maeda Y, Niino M, Takahashi T, et al Lack of KIR4.1 autoantibodies in Japanese patients with MS and NMO. Neurol—Neuroimmunol Neuroinflammation. 2016;3: e263. 10.1212/NXI.0000000000000263 PMC4959509 27489866

[29] Watanabe M, Yamasaki R, Kawano Y, Imamura S, Kira J. Anti-KIR4.1 antibodies in Japanese patients with idiopathic central nervous system demyelinating diseases. Clin Exp Neuroimmunol. 2013;4: 241–242.

[30] Nerrant E, Salsac C, Charif M, Ayrignac X, Carra-Dalliere C, Castelnovo G, et al Lack of confirmation of anti-inward rectifying potassium channel 4.1 antibodies as reliable markers of multiple sclerosis. Mult Scler. 2014;20: 1699–703. doi: 10.1177/1352458514531086 24756568 10.1177/1352458514531086

[31] Housley WJ, Pitt D, Hafler DA. Biomarkers in multiple sclerosis. Clin Immunol. 2015;161: 51–8. doi: 10.1016/j.clim.2015.06.015 26143623 10.1016/j.clim.2015.06.015

[32] Krumbholz M, Derfuss T, Hohlfeld R, Meinl E. B cells and antibodies in multiple sclerosis pathogenesis and therapy. Nat Rev Neurol. Nature Publishing Group; 2012;8: 613–23. doi: 10.1038/nrneurol.2012.203 23045237 10.1038/nrneurol.2012.203

[33] Racke MK. Disease mechanisms in MS: the potassium channel KIR4.1—a potential autoantigen in MS. Nat Rev Neurol. 2012;8: 595–6. doi: 10.1038/nrneurol.2012.193 22986435 10.1038/nrneurol.2012.193

[34] Nagelhus EA, Mathiisen TM, Ottersen OP. Aquaporin-4 in the central nervous system: cellular and subcellular distribution and coexpression with KIR4.1. Neuroscience. 2004;129: 905–13. doi: 10.1016/j.neuroscience.2004.08.053 15561407 10.1016/j.neuroscience.2004.08.053

[35] Querol L, Clark PL, Bailey M a, Cotsapas C, Cross AH, Hafler D a, et al Protein array-based profiling of CSF identifies RBPJ as an autoantigen in multiple sclerosis. Neurology. 2013;81: 956–63. doi: 10.1212/WNL.0b013e3182a43b48 23921886 10.1212/WNL.0b013e3182a43b48 PMC3888197

[36] Ayoglu B, Mitsios N, Kockum I, Khademi M, Zandian A, Sjöberg R, et al Anoctamin 2 identified as an autoimmune target in multiple sclerosis. Proc Natl Acad Sci U S A. 2016;113: 2188–93. doi: 10.1073/pnas.1518553113 26862169 10.1073/pnas.1518553113 PMC4776531

[37] Buntinx M, Vanderlocht J, Hellings N, Vandenabeele F, Lambrichts I, Raus J, et al Characterization of three human oligodendroglial cell lines as a model to study oligodendrocyte injury: morphology and oligodendrocyte-specific gene expression. J Neurocytol. 2003;32: 25–38. Available: http://www.ncbi.nlm.nih.gov/pubmed/14618099 14618099 10.1023/a:1027324230923

[38] Dale RC, Tantsis EM, Merheb V, Kumaran R-Y a, Sinmaz N, Pathmanandavel K, et al Antibodies to MOG have a demyelination phenotype and affect oligodendrocyte cytoskeleton. Neurol Neuroimmunol neuroinflammation. 2014;1: e12. 10.1212/NXI.0000000000000012 PMC4202678 25340056

[39] Lily O, Palace J, Vincent A. Serum autoantibodies to cell surface determinants in multiple sclerosis: A flow cytometric study. Brain. 2004;127: 269–279. doi: 10.1093/brain/awh031 14662514 10.1093/brain/awh031

