# Supplementary material for: Absence of antibodies against KIR4.1 in multiple sclerosis: A three-technique approach and systematic review
Source: PLoS One. 2017 Apr 17;12(4):e0175538. doi: 10.1371/journal.pone.0175538 (PMC5393569; doi:10.1371/journal.pone.0175538)
Supplement: S1 Fig — MS: multiple sclerosis, OND: other neurological diseases, HC; healthy controls. (PDF) [file pone.0175538.s002.pdf]

Author	Year publication	Country	MS	OND	HC	Analytic method	Study design	Study methodological quality
**Srivastava**	2012	Germany	N:397 Age: 35,6	N:329 Age: 45	N: 22 Age: 36	ELISA	Observational, Prospective and controlled	Strong
**Bricshawana**	2014	USA	N: 286	N: 109	N:99	ELISA	Observational, Prospective and controlled	Moderate
**Kraus**	2014	Germany	N: 47 Age: 13,6	N:44 Age: 10,55	N: 18 Age: 8,8	ELISA	Observational, Prospective and controlled	Moderate
**Nerrant**	2014	France	N: 268 Age: 46,7	N:46 Age: 45,4	N: 45 Age: 29,6	ELISA	Observational, Prospective and controlled	Moderate
**Brill**	2015	Italy	N: 131 Age: 38,1	N:61 Age: 38,8	N: 73 Age: 30,8	ELISA	Observational, Prospective and controlled	Strong
**Watanabe**	2013	Japan	N:180	N:126	N:49	ELISA	Observational, Prospective and controlled	Weak
**Marignier**	2014	France	N:177	N:56	N:20	CBA	Observational, Prospective and controlled	Weak
**Marnetto**	2015	Italy	N:27	-	N:15	ELISA	Observational, Prospective and controlled	Weak
**Malyavantham**	2015	USA	N:572	N:82	N:315	ELISA	Observational Prospective and controlled	Strong
**Navas**	2016	Spain	N:108	N:64	N:13	ELISA	Observational, Prospective and controlled	Moderate
**Chastre**	2016	USA	N:86	-	N:51	ELISA	Observational, Prospective and controlled	Moderate
**Kaya**	2015	Turkey	N:31	-	N:14	ELISA	Observational Prospective and controlled	Weak
**Higuchi**	2016	Japan	N:57	N:40	N:50	ELISA and LIPS	Observational Prospective and controlled	Weak
