# Supplementary figures and images for: Absence of antibodies against KIR4.1 in multiple sclerosis: A three-technique approach and systematic review
Source: PLoS One. 2017 Apr 17;12(4):e0175538. doi: 10.1371/journal.pone.0175538 (PMC5393569; doi:10.1371/journal.pone.0175538)

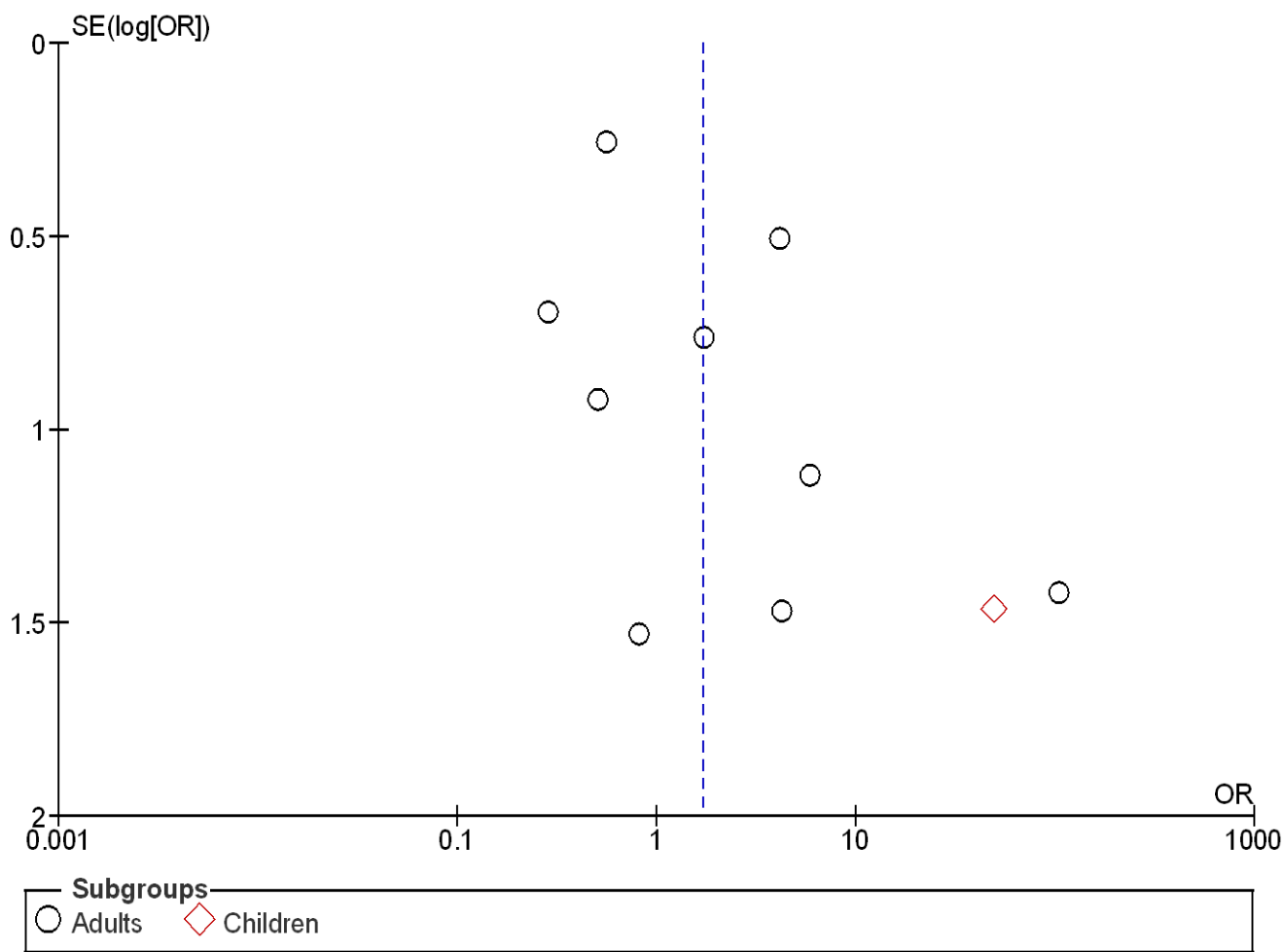

Supplement: S2 Fig — The funnel plot detects a publication bias, with smaller studies showing no-association with anti-KIR4.1 and MS. (PDF) [file pone.0175538.s003.pdf]
