# Supplementary material for: Absence of antibodies against KIR4.1 in multiple sclerosis: A three-technique approach and systematic review
Source: PLoS One. 2017 Apr 17;12(4):e0175538. doi: 10.1371/journal.pone.0175538 (PMC5393569; doi:10.1371/journal.pone.0175538)
Supplement: S1 Supplementary Methods — (PDF) [file pone.0175538.s004.pdf]

SUPPLEMENTARY METHODS – SURFACE EXPRESSION OF KIR4.1

KIR4.1-transfected MO3.13 cells (Cedarlane Labs, Canada) were used for ICC experiments. The MO3.13 cells were cultured in 60mm culture plates with poly-D-Lysine-coated coverslips and transfected as described in the Methods section. After 24 hours, cells were washed with PBS. To detect only surface KIR4.1, MO3.13 cells were incubated live with sera from patients and controls with highest OD in the KIR4.1 ELISA and a commercial antibody recognizing the 93-106 epitope of the extracellular loop of KIR4.1 (Alomone Labs, APC-165), both diluted 1:100 in growth medium. One hour later cells were washed with PBS, fixed with 4% paraformaldehyde and blocked with 5% rabbit serum in PBS for 1 hour. After 1 hour, cells were incubated with Alexa-Fluor anti-human 594 and anti-rabbit 488 (Thermo-Scientific, Spain) secondary antibodies diluted 1:500. Finally, coverslips were washed and mounted using Vectashield with DAPI (Vector Labs, UK).

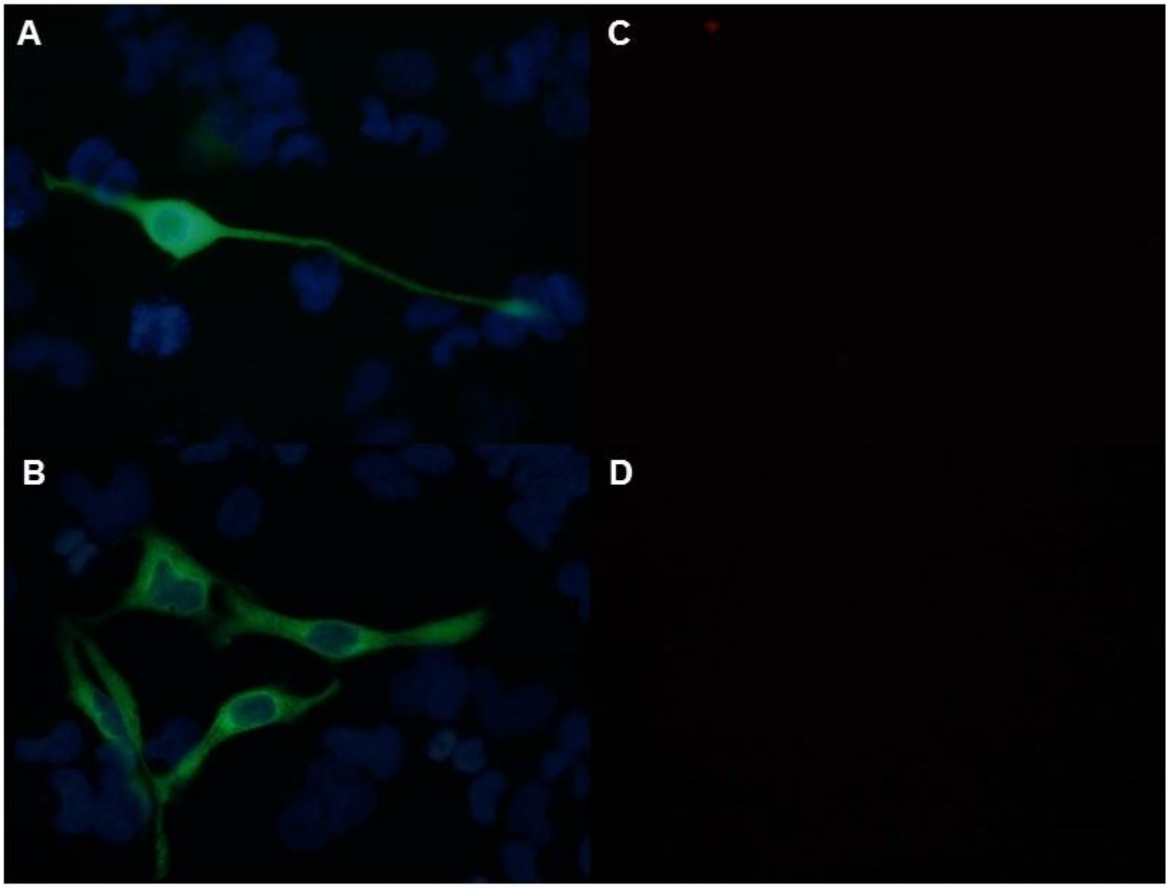

Surface expression of KIR4.1 in MO3.13 cells. Incubation of a commercial antibody recognizing the extracellular loop of KIR4.1 with live cells and fixing the cells afterwards detected a homogeneous expression of KIR4.1 in the surface of the cell (A-B). Unspecific incorporation of the primary or secondary antibodies to the cytoplasm upon fixation is ruled out since the same pattern of staining was not seen with those patient's (C) and control's (D) sera with highest KIR4.1 ELISA OD. Figures A and B are a composition of 2 images (channel for green fluorescence

and channel for blue fluorescence). This composition has not been made with red channel to highlight the complete absence of staining.
