# Supplementary material for: Absence of antibodies against KIR4.1 in multiple sclerosis: A three-technique approach and systematic review
Source: PLoS One. 2017 Apr 17;12(4):e0175538. doi: 10.1371/journal.pone.0175538 (PMC5393569; doi:10.1371/journal.pone.0175538)
Supplement: S1 Table — Epidemiological data other than gender were unavailable for 15 disease controls and average age reflects that of the ones in which information was available. MS: multiple sclerosis; HC: Healthy controls; OND: Other neurological diseases; RR: relapsing-remitting; SP: secondary progressive; PP: primary progressive. (DOCX) [file pone.0175538.s005.docx]

|  | | | MS | HC | | OND | |
| --- | --- | --- | --- | --- | --- | --- | --- |
|  | | |  |  | |  | |
| **N** | | | 108 | 13 | | 64 | |
| **Gender (female)** | | | 76 (70,3) | 8 (61,5) | | 37 (57,8) | |
| **Mean Age (range)** | | | 45,5 (22-69) | 50,3 (26-80) | | 64 (20-92) | |
| **MS-Subtype** | | |  |  | |  | |
|  | **RR-MS** | 99 (91,7) | | | NA | | NA |
|  | **SP-MS** | 4 (3,7) | | | NA | | NA |
|  | **PP-MS** | 5 (4,6) | | | NA | | NA |
| **Disease-modifying therapy** | | | 102 (94,4) | NA | | NA | |
|  | | |  |  | |  | |

**Supplementary Table 2**. Basic demographic features of patients with MS and controls included in the study. Epidemiological data other than gender were unavailable for 15 disease controls and average age reflects that of the ones in which information was available. MS: multiple sclerosis; HC: Healthy controls; OND: Other neurological diseases; RR: relapsing-remitting; SP: secondary progressive; PP: primary progressive.
